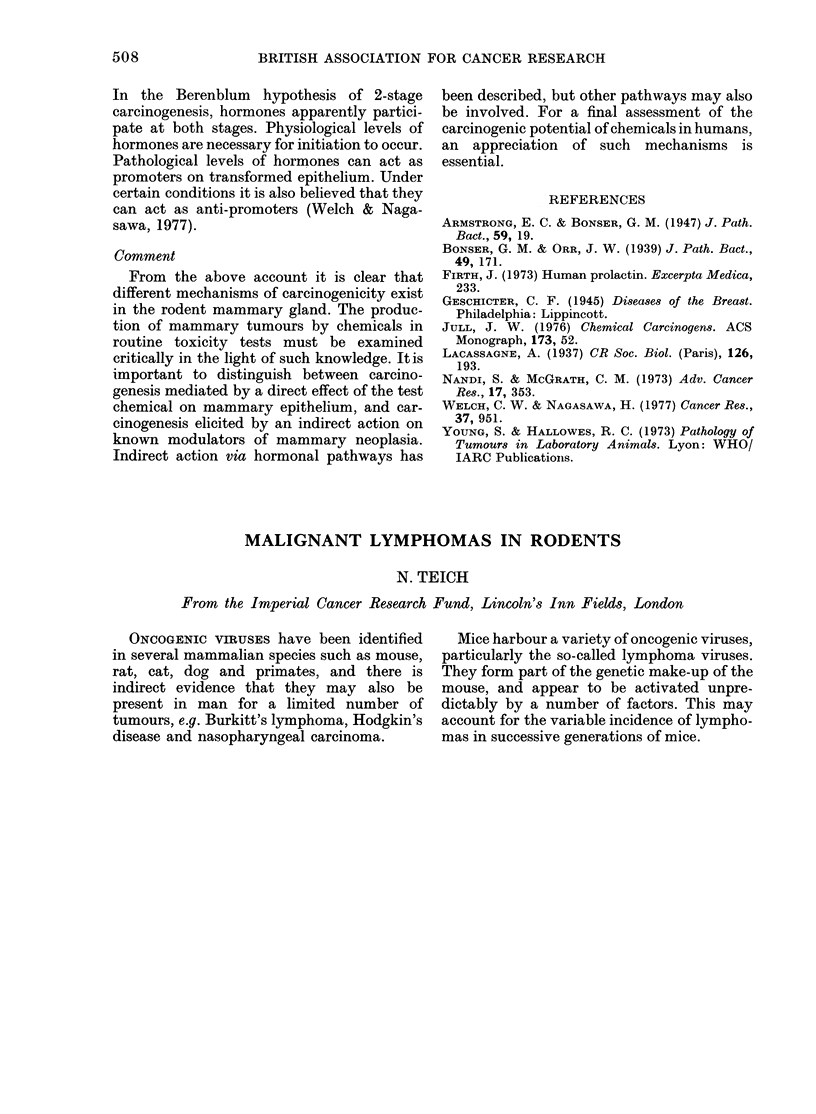# Malignant Lymphomas in Rodents

**Published:** 1980-03

**Authors:** N. Teich


### MALIGNANT LYMPHOMAS IN RODENTS

N. TEICH

*From the Imperial Cancer Research Fund, Lincoln's Inn Fields, London*

ONCOGENIC VIRUSES have been identified in several mammalian species such as mouse, rat, cat, dog and primates, and there is indirect evidence that they may also be present in man for a limited number of tumours, *e.g.* Burkitt's lymphoma, Hodgkin's disease and nasopharyngeal carcinoma.

Mice harbour a variety of oncogenic viruses, particularly the so-called lymphoma viruses. They form part of the genetic make-up of the mouse, and appear to be activated unpredictably by a number of factors. This may account for the variable incidence of lymphomas in successive generations of mice.